# Responses to GLP‐1RA in White and Black Adults With Obesity: Insights From Generalized Additive Mixed Models of EHR Data

**DOI:** 10.1002/oby.70180

**Published:** 2026-04-01

**Authors:** Jordan H. Mallette, Joshua S. Speed, Seth T. Lirette, Daniel Moore, John S. Clemmer

**Affiliations:** ^1^ Department of Physiology and Biophysics University of Mississippi Medical Center Jackson Mississippi USA; ^2^ Department of Data Science University of Mississippi Medical Center Jackson Mississippi USA

**Keywords:** GLP‐1 agonist, obesity, racial disparities, weight loss

## Abstract

**Objective:**

This study examined racial differences in weight loss and clinical response to glucagon‐like peptide‐1 receptor agonist (GLP‐1RA) therapy among adults with obesity using real‐world data.

**Methods:**

We retrospectively analyzed our institution's Research Data Warehouse (RDW) containing deidentified data from electronic health records (EHRs) from adults aged 18 years and older. EHR data from the University of Mississippi Medical Center RDW were analyzed for 7214 adults (2182 White; 5032 Black) prescribed injectable GLP‐1RA between 2015 and 2025. Inclusion criteria were BMI ≥ 30 kg/m^2^ and ≥ 1 GLP‐1RA. Longitudinal changes in patient data were modeled using generalized additive mixed models.

**Results:**

At 2.5 years, White individuals experienced significantly greater body weight loss than Black individuals: −7.1% (95% CI −8.0%, −6.2%) versus −4.9% (95% CI −5.4%, −4.3%), as well as significantly larger reductions in estimated glomerular filtration rate, systolic blood pressure, and HbA1c. Semaglutide and tirzepatide were associated with the greatest improvements. Renal function decline was attenuated in both groups after therapy.

**Conclusions:**

In a large, diverse EHR cohort, GLP‐1RA therapy was associated with significant weight, glycemic, and renal benefits that were attenuated in Black adults. These findings highlight the urgent need to understand mechanisms contributing to variable GLP‐1RA outcomes among different populations.

## Study Importance

- What is already known?
  ○ Clinical trials demonstrate substantial weight loss and metabolic improvements with GLP‐1RA therapies, yet most studies underrepresent Black adults and rarely assess racial differences in treatment response despite higher prevalences of obesity and uncontrolled diabetes in this population.
- What does this study add?
  ○ Using data from routine clinical practice (EHR), Black adults lost ~30% less weight with GLP‐1RA and had less improvement in blood pressure, HbA1c, and estimated glomerular filtration rate than White counterparts.
- How might these results change the direction of research or focus of clinical practice?
  ○ Whether responses to GLP‐1RA therapy may vary across racial and socioeconomic groups is unknown, highlighting a critical gap in understanding disparities in obesity treatment efficacy. These data underscore the need to test GLP‐1RA across diverse populations with obesity and warrant further investigation into the underlying causes of racial disparities in outcomes of routine clinical practice.

## 1 Introduction

Obesity affects over 100 million adults and poses a serious health and economic burden in the United States [1]. Over 48% of adults are predicted to have obesity by 2030 [2] and every two in three adults by 2050 [3]. With the expected rise in the number of adults with obesity, effective weight management therapies are critical to reduce the growing burden of obesity‐related hypertension, cardiovascular disease, and kidney disease. A meta‐analysis of over 10 million adults in 32 countries found adults with a body mass index (BMI) > 40 kg/m^2^ had nearly a threefold higher risk of all‐cause mortality (HR 2.8) compared with those of normal weight [4]. Management of obesity includes lifestyle interventions and dietary modifications, but these have shown little success in long‐term weight reduction [5, 6, 7]. If successful reduction in body weight (≥ 10%) is achieved, these patients have significant improvements in cardiovascular morbidity and mortality, in part through the resultant benefits in blood pressure (BP), cardiac demand, and glycemic control, as well as renal health [8, 9].

New pharmacotherapeutic interventions including glucagon‐like peptide‐1 (GLP‐1) receptor agonists (GLP‐1RA) offer a new avenue for weight loss and improved obesity‐mediated morbidity. Dulaglutide is currently approved only for type 2 diabetes (T2D), while semaglutide and liraglutide are approved for both T2D and obesity. In 2023, tirzepatide became the first dual GLP‐1RA and glucose dependent insulinotropic polypeptide receptor agonist approved for chronic weight management, with weight loss of up to 20% [10, 11]. However, racial differences in outcomes with this relatively new class of drugs for weight management are understudied.

To date, clinical trials evaluating racial differences in GLP‐1RA response have largely involved older formulations and have typically lasted less than 1 year [12]. In earlier trials testing semaglutide in T2D patients, both White and Black patients had modest but similar weight loss (only 20%–22% achieving ≥ 10% reduction after 6 months) [12]. However, racial differences in long‐term (≥ 2 years) trials of high‐dose semaglutide or tirzepatide in Black patients are unclear.

Despite a higher burden of T2D and obesity in Black or African Americans [13], GLP‐1RA controlled clinical trials include < 10% of Black individuals [14]. Moreover, two recent studies have reported Black individuals are significantly less likely to receive GLP‐1RA in real‐world practice [15]. Thus, this raises important questions about whether outcomes reported in controlled clinical GLP‐1RA trials extend to individuals from diverse racial backgrounds in a real‐world setting. Using electronic health records (EHRs) from the University of Mississippi Medical Center (UMMC), the goal of this study was to investigate whether the use of GLP‐1RA in Black adults results in the same weight loss benefits as reported in White individuals.

## 2 Methods

### 2.1 Study Population

**FIGURE 1:**
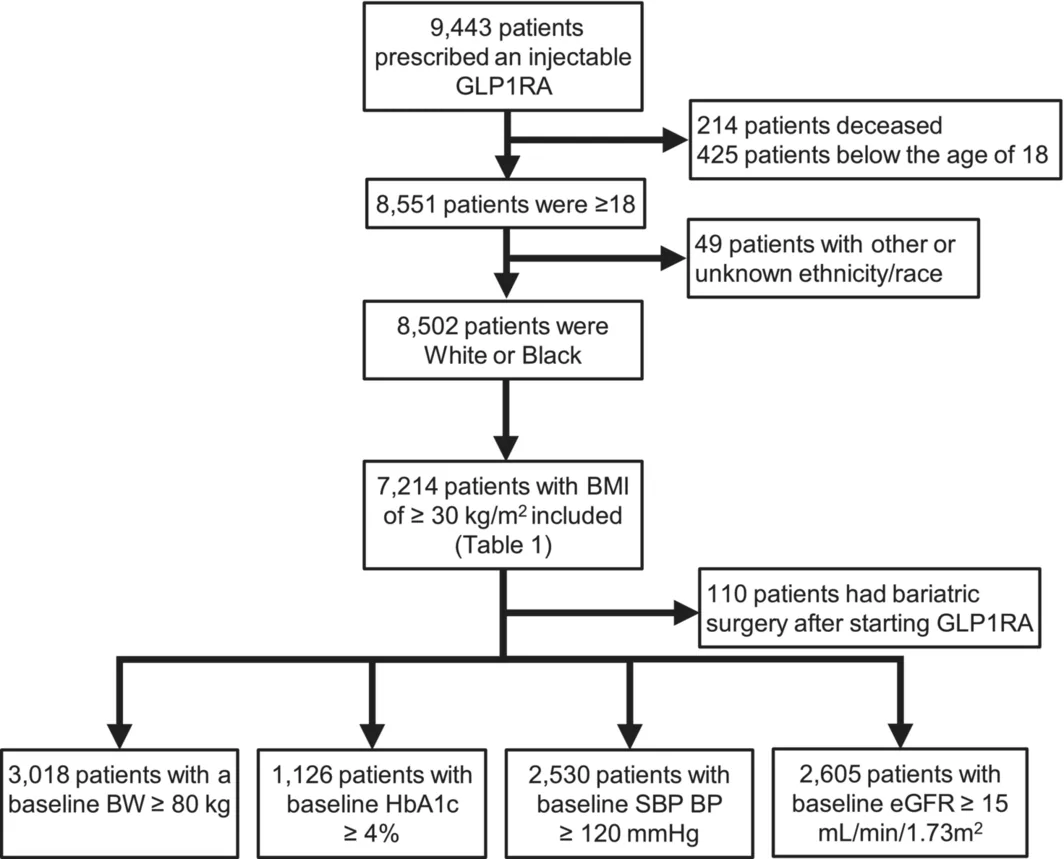
Inclusion criteria for patients taking GLP‐1RA within the Research Data Warehouse. Sample sizes represent subsets of the overall cohort with available data for each respective outcome. GLP‐1RA indicates glucagon‐like peptide‐1 receptor agonist; BW, body weight; eGFR, estimate glomerular filtration rate; HbA1c, hemoglobin A1c; SBP, systolic blood pressure.

The UMMC Research Data Warehouse (RDW) contains deidentified EHR data from approximately 1.4 million unique patients encompassing more than 62 million clinical encounters. All RDW data are date‐shifted and fully deidentified in compliance with the Health Insurance Portability and Accountability Act (HIPAA). Pursuant to 45 CFR 46, analyses of RDW data do not meet the regulatory definition of human subjects research and are exempt from institutional review board (IRB) oversight. GLP‐1RA formulations analyzed included dulaglutide (0.75–4.5 mg), semaglutide (0.25–2.4 mg), liraglutide (0.6–1.8 mg), and tirzepatide (2.5–15 mg). Eligible patients were White or Black adults with obesity, defined as BMI ≥ 30 kg/m^2^ with at least one clinical visit associated with a GLP‐1RA formulation listed earlier. Baseline BMI was required to be recorded within 1 year prior to treatment initiation. While not criteria for Table 1 (cross‐sectional/baseline summary of the population), for each longitudinal outcome, patients were only included if they had at least two measurements both before and after GLP‐1RA initiation for each generalized additive mixed model (GAMM). Also, patients were excluded if they had a history of bariatric surgery or lacked a qualifying baseline measurement for outcomes of interest. Additional outcome‐specific exclusions were applied to ensure realistic clinical ranges and model stability: baseline body weight < 80 kg, estimated glomerular filtration rate (eGFR) < 15 mL/min/1.73 m^2^, hemoglobin A1c (HbA1c) < 4%, or systolic blood pressure (SBP) < 120 mmHg. Data occurring more than 5 years from GLP‐1RA initiation were not analyzed. Among the 9443 patients who were prescribed GLP‐1RA in the RDW, 7214 were Black or White and fit the inclusion criteria (Figure 1). Sample sizes varied across outcome‐specific analyses because not all patients had available pre‐ and post‐treatment measurements for each outcome.

**TABLE 1:** Baseline characteristics of patients with obesity in the Research Data Warehouse who were administered GLP‐1RA.

	White	Black
*N*	2182	5032
Female (%)	64	79*
Age, years	54 ± 14	49 ± 13*
BMI, kg/m^2^	40 ± 8	43 ± 9*
Weight, kg	114 ± 25	121 ± 29*
Follow‐up, years	2.1 ± 1.5	2.2 ± 1.4
Office visits^a^	14 ± 15	16 ± 17
GLP‐1RA prescriptions^a^	26 ± 21	28 ± 21
SBP, mmHg	138 ± 12	140 ± 14*
HbA1c, %	7.88 ± 2.0	8.41 ± 2.4*
eGFR, mL/min/1.73 m^2^	85 ± 25	82 ± 28
Semaglutide (%)	53	52
Tirzepatide (%)	14	10*
Liraglutide (%)	20	20
Dulaglutide (%)	13	18*
Medicaid (%)	10	18*
T2D (%)	67	74*
HTN (%)	83	89*
Dyslipidemia (%)	36	33*
AKI (%)	10	13*
CKD (%)	20	24*
ESRD (%)	1	4*
HF (%)	12	15*
CAD (%)	22	15*
MI (%)	7	6

Abbreviations: AKI, acute kidney injury; CAD, coronary artery disease; CKD, chronic kidney disease; ESRD, end stage renal disease; FU, follow‐up; HbA1c, hemoglobin A1c; HF, heart failure; HTN, hypertension; MI, history of previous myocardial infarction; SBP, systolic blood pressure; T2D, type 2 diabetes.

^a^ GLP‐1RA prescriptions included visits associated with a prescription plus refills prescribed. GLP‐1RA formulation % represents the majority of prescriptions for each patient.

* *p* < 0.05 versus White.

Baseline comorbidities including T2D, hypertension, dyslipidemia, coronary artery disease, acute kidney injury, chronic kidney disease, end stage renal disease, heart failure, and myocardial infarction were identified using SNOMED‐CT diagnostic codes (Table S1). Comorbidity diagnoses were only considered if recorded within 1 year from the date of the first GLP‐1RA or any time before first prescription.

### 2.2 Outcomes

The prespecified primary outcomes of the study were (1) percent change in body weight over time following GLP‐1RA initiation and (2) achievement of clinically meaningful weight loss, defined as ≥ 10% reduction from baseline body weight. Percent weight change was calculated relative to baseline weight measured within the year prior to GLP‐1RA initiation. Secondary outcomes included longitudinal changes in HbA1c, SBP, and kidney function. Kidney outcomes were assessed as both change in eGFR and differences in eGFR slope (normalized per year) before versus after GLP‐1RA initiation to evaluate treatment‐associated change in the rate of kidney function decline. eGFR was estimated from plasma creatinine values using the Chronic Kidney Disease Epidemiology Collaboration (CKD‐EPI) 2021 equation without race coefficient.

### 2.3 Statistical Analysis

All analyses were performed in RStudio (version 2025.05.1+513). For the baseline analysis in Table 1, variables were automatically classified as continuous or categorical based on data type; binary (0/1) variables were treated as categorical regardless of storage type. Continuous variables were reported as mean ± standard deviation (SD). Between‐group comparisons were conducted using a Wilcoxon rank sum test. For categorical variables, results were summarized as percentages. Group differences were evaluated using the chi‐square test of independence. All statistical tests were two‐tailed, and *p* values < 0.05 were considered statistically significant.

To evaluate longitudinal changes in body weight, BP, eGFR, and HbA1c, we used the GAMMs with a random intercept and slope for time at the patient level. Models were fit separately for time points before and after the initiation of GLP‐1RA. Patient‐level data consisted of repeated measurements before and after initiation of GLP‐1RA therapy (defined as year 0). All data that were after GLP‐1RA initiation and within 3 months of the last GLP‐1RA recorded were included in the post model. Each model treated years as the primary continuous time variable, allowing for nonlinear trajectories of change to be estimated separately for White and Black patients using race‐specific smooth functions of time. For change in body weight, for example:

$$\begin{array}{c} {\text{Weight change}}_{ij}=f_{ra}\left({Years}_{ij}\right)++X_{ij}+b_{0i}+b_{1i}{Years}_{ij}+{𝑖}_{ij} \end{array}$$

where 𝑓_𝑟𝑎_ denotes a thin‐plate regression spline for time stratified by race, 𝑋_𝑖𝑗_ represents covariates, 𝑏_0𝑖_ and 𝑏_1𝑖_ are patient‐specific random intercepts and slopes (accounting for within‐subject correlation), and 𝜀_𝑖𝑗_ is the residual error. Models for the pretreatment period were adjusted for age, sex, race, diabetes status, and Medicaid enrollment. For post‐treatment, models were controlled for age, sex, race, diabetes status, Medicaid enrollment, BMI, outcome‐specific baseline values (body weight, HbA1c, SBP, or eGFR), and GLP‐1RA frequency and formulation. A race‐specific smooth term for time was included with up to 10 basis functions. Continuous covariates were modeled flexibly using penalized smoothing splines, while categorical variables were entered as fixed‐effect factors. All models were estimated using restricted maximum likelihood. Smooth term estimates and their 95% confidence intervals (CI) were derived using Bayesian posterior covariance estimates and provided in the online Supporting Information (Figures S1–S4). Parametric coefficients from the GAMMs are provided in Table S2. The outcome of the GAMMs was change in body weight (%), HbA1c (%), SBP (mmHg), and eGFR (mL/min/1.73 m^2^) over the treatment period. Beta coefficients (*β*) from the GAMMs represent the vertical difference in the outcome. Follow‐up duration varied across patients. To facilitate interpretation of model results, adjusted marginal estimates (margins) were derived from the fitted GAMMs. Adjusted race‐specific mean outcomes at 1, 2.5, and 5 years were obtained from the fitted GAMMs by evaluating the model at fixed covariate values. Differences between White and Black patients at each time point were estimated using linear contrasts of the fitted model, with standard errors obtained from the model‐based covariance matrix [16]. Two‐sided Wald tests were used to derive 95% CI and *p* values (Table 2).

**TABLE 2:** GAMM adjusted responses to GLP‐1RA at 1, 2.5, and 5 years by race.

Time since GLP‐1RA initiation	White, change (95% CI)	Black, change (95% CI)	White versus Black, *p*
Body weight (%)			
1 year	−0.060 (−0.065, −0.055)	−0.045 (−0.049, −0.041)	< 0.001
2.5 years	−0.079 (−0.089, −0.070)	−0.057 (−0.063, −0.051)	< 0.001
5 years	−0.126 (−0.146, −0.106)	−0.105 (−0.117, −0.093)	0.0796
HbA1c (%)			
1 year	−1.235 (−1.472, −0.998)	−1.121 (−1.311, −0.931)	0.2559
2.5 years	−1.316 (−1.601, −1.032)	−1.003 (−1.215, −0.790)	0.0224
5 years	−1.870 (−2.549, −1.190)	−1.207 (−1.783, −0.630)	0.1310
Blood pressure (mmHg)			
1 year	−9.038 (−10.177, −7.898)	−6.186 (−7.102, −5.269)	< 0.001
2.5 years	−10.015 (−11.358, −8.672)	−6.297 (−7.326, −5.269)	< 0.001
5 years	−11.643 (−13.904, −9.381)	−9.373 (−11.380, −7.365)	0.115
eGFR (mL/min/1.73 m^2^)			
1 year	−0.727 (−2.002, 0.548)	−2.234 (−3.291, −1.176)	0.00167
2.5 years	−2.676 (−4.157, −1.194)	−4.639 (−5.776, −3.502)	0.00282
5 years	−5.923 (−8.252, −3.595)	−8.648 (−10.183, −7.112)	0.02898

*Note:* Values represent adjusted mean change and 95% CI estimated from generalized additive mixed models (GAMMs) with race‐specific smooth functions of time, random intercepts and slopes, and adjustment for age, sex, diabetes status, Medicaid enrollment, baseline BMI, baseline values, and GLP‐1RA formulation and frequency. Continuous covariates were fixed at their sample means, and categorical covariates were fixed at their model values unless otherwise specified. *p* values correspond to model‐based White–Black contrasts at each time point derived using the GAM lpmatrix and posterior covariance of the model coefficients, with standard errors computed from linear contrasts and two‐sided Wald tests. eGFR indicates estimated glomerular filtration rate (CKD‐EPI 2021 equation), and HbA1c indicates hemoglobin A1c.

Cox proportional hazard regression was used to estimate the association between covariates and the time to achieving ≥ 10% weight loss following GLP‐1RA initiation controlling for covariates listed earlier. Time‐to‐event was defined as the earliest post‐treatment visit at which ≥ 10% weight reduction from baseline body weight was observed. Patients not reaching this threshold were censored at their last follow‐up. Kaplan–Meier curves stratified by race, drug frequency, and drug type were plotted to visualize unadjusted time‐to‐event distributions.

## 3 Results

Baseline characteristics of the study population are presented in Table 1. The total analyzed cohort included 2182 White and 5032 Black adults. Compared with White individuals, Black patients were on average 5 years younger and 7 kg heavier. Black individuals were more likely to be female (79% vs. 64%) and enrolled in Medicaid (18% vs. 10%). The prevalence of T2D (74% vs. 67%) was significantly greater among Black adults compared with their White counterparts, yet fewer Blacks had incidence of dyslipidemia as compared to White patients (33% vs. 36%). Prevalence of hypertension (89% vs. 83%) was significantly higher in Black compared to White individuals. No significant differences were observed between racial groups in the mean number of GLP‐1RA prescriptions or duration of follow‐up. Semaglutide and liraglutide were the most often prescribed, with no differences in rates between Black and White groups. However, Black adults were prescribed tirzepatide less frequently (10% vs. 14%) and dulaglutide (18% vs. 13%) more frequently than White counterparts. Baseline prevalence of acute kidney injury (13% vs. 10%), chronic kidney disease (24% vs. 20%), and end‐stage renal disease (4% vs. 1%) was also significantly higher among Black compared with White individuals. Median follow‐up was approximately 2 years for both groups (Table 1).

### 3.1 Change in Body Weight in Response to GLP‐1RA

**FIGURE 2:**
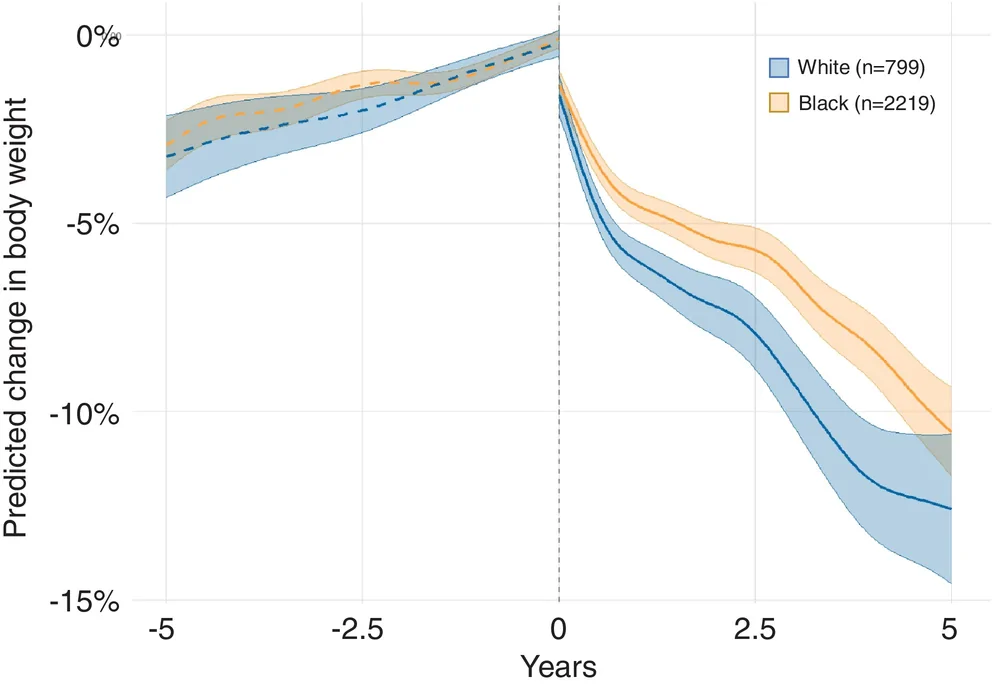
Race‐specific changes in body weight before and during GLP‐1RA therapy. Predicted nonlinear trajectories of percent body weight change from generalized additive mixed models (GAMMs) stratified by White (blue) and Black (orange) race. Both pre (years −5 to 0) and post (years 0–5) models include a race‐specific smooth term for time and adjust for age, sex, diabetes, Medicaid insurance, and (in the post‐treatment model only) GLP‐1RA type and frequency, baseline weight, and baseline BMI. Lines represent predicted means; shaded bands indicate 95% CI.

The GAMM constructed for body weight reports percent change relative to baseline (Day 0) and was modeled as a nonlinear smooth function of time since GLP‐1RA initiation, with covariate parameters (*β*) referring to vertical shifts of the change. There was a gradual increase in body weight before the first GLP‐1RA prescription that was similar between races (Figure 2). Age, diabetes, and Medicaid status were not significant predictors of weight gain before therapy.

After therapy onset (*n* = 46,957 observations across 3018 patients), the GAMM predicted that younger age, lower starting weight, Black race, and higher BMI were significantly associated with less weight loss (Figure S1). Compared with females, males had 1.3% less weight reduction than females over time after adjusting for other variables in the models (Table S2). Diabetes status predicted slightly less weight loss (*β* = 0.57% ± 0.27%, *p* = 0.033), while Medicaid insurance was not significant (Table S2). Among GLP‐1RA formulations, semaglutide and tirzepatide were associated with the largest reduction in body weight represented in the GAMM shown in Table S2 (*β* = −1.06% ± 0.23% and −1.24% ± 0.38%, respectively; both *p* < 0.001).

**FIGURE 3:**
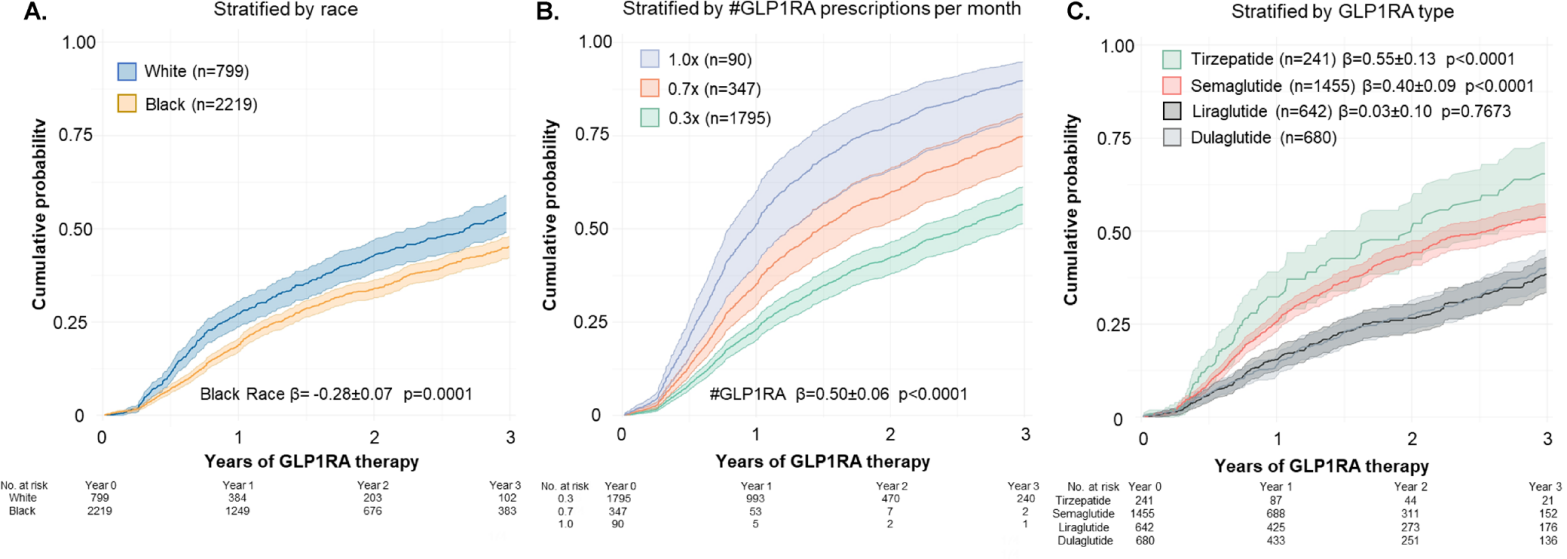
Cumulative probability of significant weight loss (≥ 10%) during GLP‐1RA therapy. Cox proportional hazard regression model of the estimated time to achieving ≥ 10% weight loss following GLP‐1RA initiation controlling for sex, race, diabetes status, Medicaid enrollment, BMI, baseline values, and GLP‐1RA frequency and formulation. Time‐to‐event was defined as the earliest post‐treatment visit at which ≥ 10% weight reduction from baseline body weight was observed. Kaplan–Meier curves were (A) stratified by race, (B) stratified by visits associated with GLP‐1RA prescriptions per month, or (C) stratified by GLP‐1RA type (in comparison to dulaglutide). Relevant model parameter estimates (*β*) are shown.

While Black patients showed a significant weight loss at 1 and 2.5 years after GLP‐1RA initiation (−3.7% [95% CI −3.9%, −3.4%] and −4.9% [95% CI −5.4%, −4.3%], respectively), this was ~30% less than the weight loss seen in White patients (−5.2% [95% CI −5.6%, −4.7%] and −7.1% [95% CI −8.0%, −6.2%], respectively) (Table 2). Black individuals also had a significantly lower probability of losing at least 10% body weight (Figure 3A) compared to White individuals. In contrast, more frequent GLP‐1RA dosing (Figure 3B) and use of tirzepatide or semaglutide (Figure 3C) were associated with a significantly greater probability of achieving at least 10% body weight loss.

### 3.2 Change in HbA1c

**FIGURE 4:**
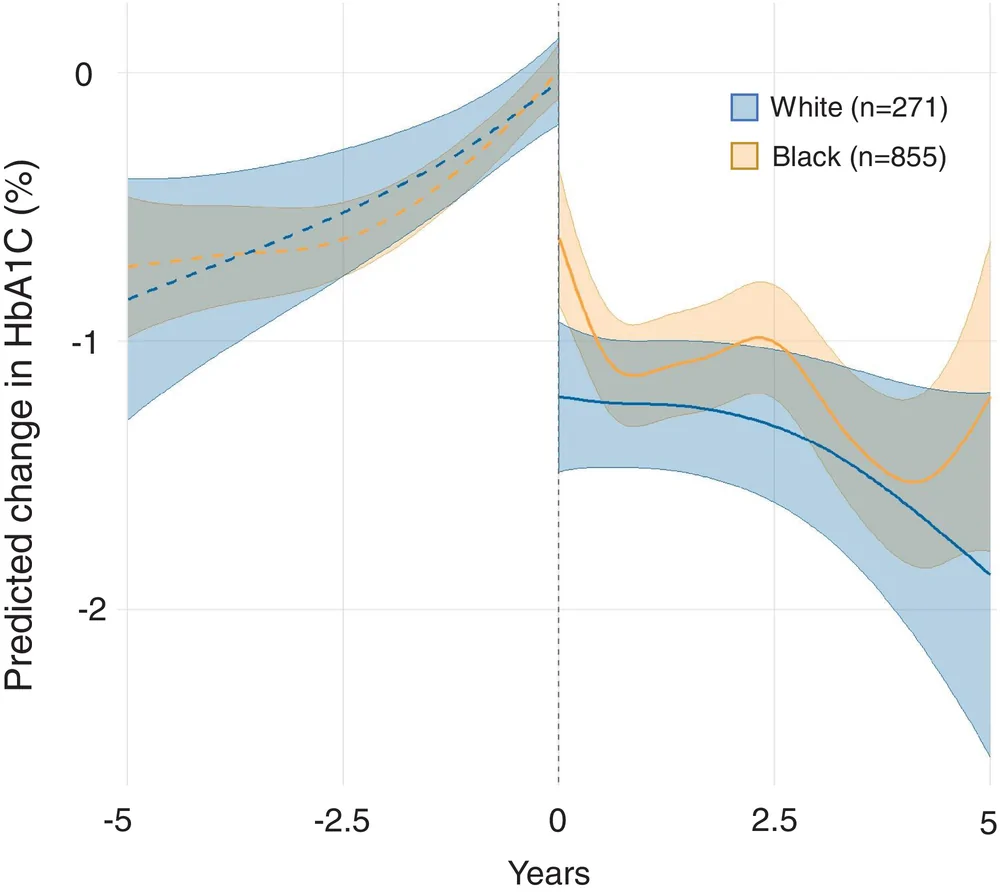
Race‐specific changes in hemoglobin A1c (HbA1c) before and during GLP‐1RA therapy. Predicted nonlinear changes in HbA1c from generalized additive mixed models (GAMMs) stratified by White (blue) and Black (orange) race. Both pre (years −5 to 0) and post (years 0–5) models include a race‐specific smooth term for time and adjust for the same covariates listed in Figure 2. Lines represent predicted means; shaded bands indicate 95% CI.

HbA1c at baseline was significantly higher in the Black group and, as expected, was associated with prevalence of T2D (Table 1). Prior to treatment, none of the parametric covariates was significantly associated with HbA1c change. However, HbA1c significantly increased similarly in both races during the 5 years prior to GLP‐1RA and revealed significant, race‐specific, nonlinear HbA1c trajectories (Figure 4). At 2.5 years, White patients had a significantly greater reduction in HbA1c as compared to Black counterparts (−1.1% [95% CI −1.4%, −0.9%] vs. −0.8% [95% CI −1.0%, −0.7%]), while there were no statistical differences at 1 or 5 years (Table 2). Medicaid enrollment was associated with higher post‐treatment HbA1c, while sex was not a significant factor (Table S2). In addition to White race, older age (*p* = 0.037), higher GLP‐1RA dose (*p* = 0.006), semaglutide/tirzepatide use, and higher baseline HbA1c (*p* < 0.001) were all significant predictors of HbA1c reduction (Figure S2 and Table S4).

### 3.3 Change in Systolic Blood Pressure

**FIGURE 5:**
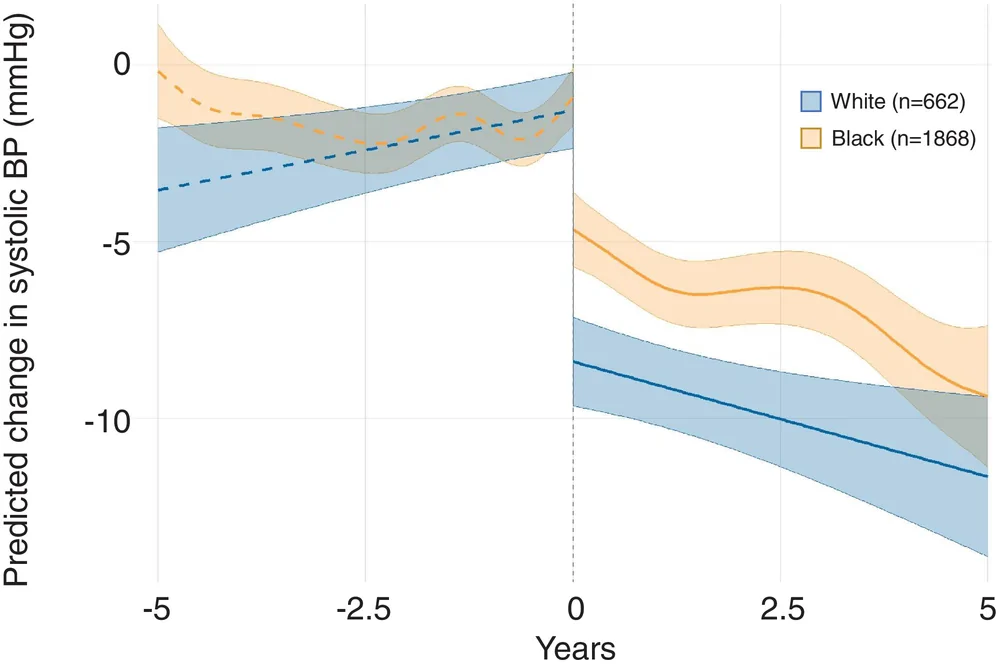
Race‐specific changes in systolic blood pressure before and during GLP‐1RA therapy derived from generalized additive mixed models. Dashed lines indicate before therapy, and solid lines indicate during GLP‐1RA therapy. The dashed and solid lines represent the model estimated smooth term for systolic blood pressure, with shading indicating 95% CI. The blue line represents White adults, and the orange line represents Black adults.

Baseline SBP was slightly but significantly higher in Black individuals versus White counterparts (Table 1; 140 ± 14 vs. 138 ± 12 mmHg, respectively). Before GLP‐1RA, males exhibited significantly higher BP compared to females (*β* = 1.47 ± 0.5 mmHg, *p* = 0.007). There were no statistically significant effects of diabetes status, age, or Medicaid status on BP prior to treatment (Figure 5). After therapy initiation (*n* = 39,118 observations across 2530 individuals), both races had a significant reduction in average SBP, with significantly lower SBP seen in the White group for years 1 and 2.5 (−7.6 mmHg [95% CI −8.5, −6.8] and −8.6 mmHg [95% CI −9.7, −7.5], respectively) as compared to Black counterparts (−4.8 mmHg [95% CI −5.3, −4.2] and −4.9 mmHg [95% CI −5.6, −4.2], respectively) (Table 2). The GAMM predicted that male sex (*β* = 1.8 ± 0.5 mmHg, *p* < 0.001) and presence of diabetes (*β* = 1.3 ± 0.6 mmHg, *p* = 0.040) were each associated with significantly higher BP during GLP‐1RA therapy (Table S2). Compared to dulaglutide, semaglutide (*β* = −2.0 ± 0.6 mmHg, *p* < 0.001) and tirzepatide (*β* = −2.1 ± 0.8 mmHg, *p* = 0.014) were associated with a significantly greater reduction in BP.

### 3.4 Change in Estimated Glomerular Filtration Rate

**FIGURE 6:**
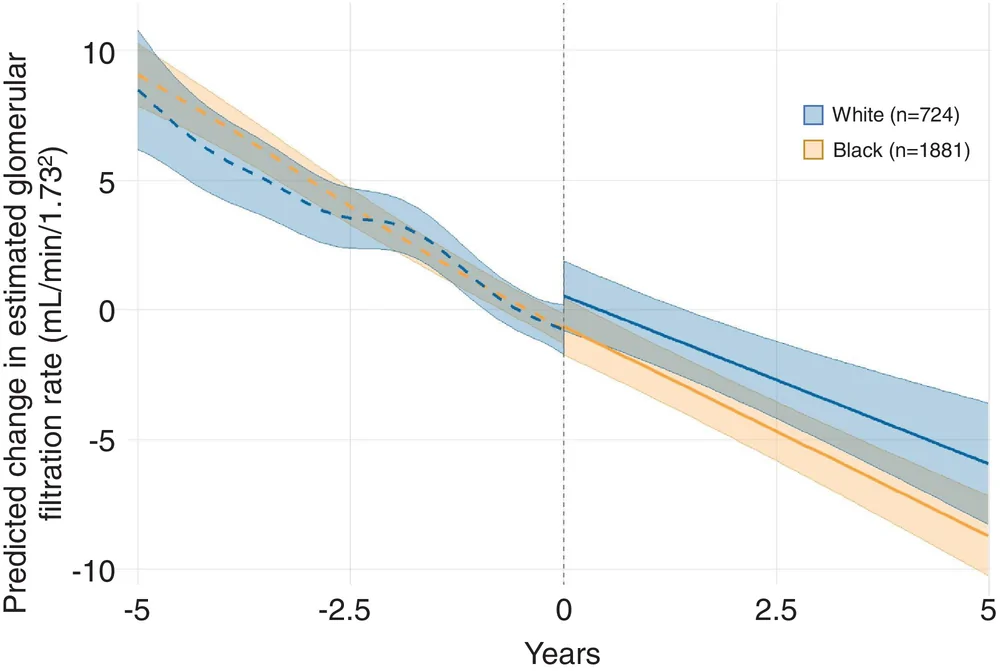
Race‐specific changes in estimated glomerular filtration rate (eGFR) before and during GLP‐1RA therapy. Predicted changes in eGFR from generalized additive mixed models (GAMMs) stratified by White (blue) and Black (orange) race. Both pre (years −5 to 0) and post (years 0–5) models are adjusted for the same covariates listed in Figure 2. eGFR values calculated with the CKD‐EPI 2021 equation using plasma creatinine. Lines represent predicted means; shaded bands indicate 95% CI.

Baseline eGFR was similar between White and Black patients (85 ± 25 vs. 82 ± 28 mL/min/1.73 m^2^) (Table 1). Before GLP‐1RA initiation, none of the included parametric covariates—sex, race, diabetes status, or Medicaid enrollment—was significantly associated with change in eGFR. In the post‐treatment period (*n* = 18,223 measurements), Medicaid enrollment (*β* = −1.27 ± 0.452 mL/min/1.73 m^2^, *p* = 0.005) was associated with a significantly lower eGFR. The change in eGFR was significantly greater in Black patients 1, 2.5, and 5 years after GLP‐1RA (−3.5 mL/min/1.73 m^2^[95% CI −4.0, −3.0], −5.9 mL/min/1.73 m^2^ [95% CI −6.6, −5.3], and −9.9 mL/min/1.73 m^2^ [95% CI −11.1, −8.7], respectively), as compared to White patients (−2.0 mL/min/1.73 m^2^ [95% CI −2.8, −1.2], −4.0 mL/min/1.73 m^2^ [95% CI −5.1, −2.8], and −7.2 mL/min/1.73 m^2^ [95% CI −9.3, −5.1], respectively) (Table 2, Figure 6).

## 4 Discussion

The current analysis of real‐world EHR data of patients from Mississippi mirrors national trends, showing that Black individuals had higher rates of T2D, hypertension, and CKD and exhibited greater body weight as compared with White individuals. In this analysis, GLP‐1RA treatment with a median of 2‐year follow‐up was associated with a significant reduction in body weight, supporting its use clinically for weight management. However, Black patients experienced ~30% less body weight reduction compared to White patients, which persisted to at least 2.5 years after GLP‐1RA (Table 2). Subsequently, Black individuals experienced less improvement in other clinical measurements, including BP, HbA1c, and eGFR, as compared to White counterparts. These real‐world data underscore the need to test GLP‐1RA efficacy as an obesity treatment across diverse populations and warrant further investigation into the underlying causes of racial disparities in outcomes of routine clinical practice.

Newer controlled GLP‐1RA trials in patients with obesity (both diabetic and non‐diabetic populations) demonstrated weight reduction ranging from 5% to 20% [17, 18, 19, 20, 21, 22]. In fact, one of the most efficacious GLP‐1RA formulations, tirzepatide, is associated with ~70% of participants losing ≥ 10% body weight in 72 weeks [17]; however, only 8% of the trial participants were Black. Our data suggest tirzepatide is also the most efficacious GLP‐1RA formulation in Black individuals. For the Black population, the GAMMs predicted −4.8% (95% CI −5.5%, −4.2%) weight loss at 1 year and −10.8% (95% CI −12.1%, −9.5%) if individuals were on GLP‐1RA for 5 years (Table S3). In the current study, Black patients, who were more likely to be on Medicaid and less likely to get tirzepatide, showed only 19% losing ≥ 10% body weight by year 1 (vs. 27% seen in Whites) (Figure 3A). While factors related to social determinants of health, such as insurance coverage, drug type and costs, and drug compliance, may explain some of this disparity, there are likely other potential mechanisms that remain unelucidated. Indeed, our current data show race still to be a significant long‐term factor in blunting weight loss, even when controlling for Medicaid insurance status, frequency of GLP‐1RA prescription, and GLP‐1RA formulation.

Real‐world efficacy of GLP‐1RA in obesity management is not well defined. In the current study, body weight loss after 2.5 years in White and Black patients was −7.1% (95% CI −8%, −6.2%) and −4.9% (95% CI −5.4%, −4.3%), respectively, with further weight loss predicted if therapy persisted for 5 years (Figure 2). In the SURMOUNT‐1 trial, tirzepatide was associated with a −15% reduction in weight after 72 weeks [17]. The STEP 8 trial, which investigated GLP‐1RA in addition to recommended changes in diet and physical activity, reported that once‐weekly semaglutide was associated with a significantly greater reduction in body weight as compared with once‐daily liraglutide (−15.8% vs. −6.4%, respectively) [23]. However, an analysis of real‐world data found semaglutide and liraglutide were only associated with a −5.1% and −2.2% change in body weight after 1 year, respectively [24]. Similarly, a study of EHR data of patients with obesity in Chicago contained a high percentage of Black individuals and indicated that high‐dose semaglutide resulted in only 5% weight loss after 12 months [25]. Thus, these data suggest controlled clinical trials may overestimate the weight loss with GLP‐1RA as compared to routine clinical practice, which could be related to low diversity.

Weight loss is associated with improvements in BP [26]. Unfortunately, racial differences in BP control during GLP‐1RA treatment have not been consistently shown in previous trials. With relatively similar weight loss (~5 kg), SUSTAIN 1–5 trials demonstrated −5 versus −6 mmHg in White and Black individuals, respectively, while the SUSTAIN 6 trial reported −6 versus −2 mmHg in White and Black individuals, respectively [12]. In our analysis, White and Black individuals both had a significant reduction in BP with GLP‐1RA, but slightly more so in White individuals (−8.6 mmHg [95% CI −9.7, −7.5] and −4.9 mmHg [95% CI −5.6, −4.2] for White and Black groups, respectively) at 2.5 years after controlling for age, sex, BMI, Medicaid, GLP‐1RA frequency, and GLP‐1RA formulation (Table 2, Figure 5). As expected, semaglutide and tirzepatide, which were associated with the greatest reductions in body weight, yielded the most improvements in BP in both White and Black groups (Table S5). This data suggests reduction in body weight is a significant driver for improvements in BP, and that the attenuated weight loss seen in Black individuals is associated with less of a BP fall during GLP‐1RA treatment.

Obesity is a significant risk factor for CKD. In the current study, Black patients had a significantly higher prevalence of CKD at baseline (Table 1). The FLOW trial, which included patients with T2D and CKD, found that semaglutide improved renal outcomes but Black individuals represented only 4% of the trial population [27]. In our analysis, Black race and Medicaid status were significant predictors of eGFR decline, suggesting that both race and socioeconomic status attenuate the renal benefits to GLP‐1RA independently (Table S2). Further research is desperately needed to evaluate long‐term renal outcomes in diverse patient populations receiving GLP‐1RA.

The mechanism for differing racial responses to GLP‐1RA is currently unknown. Race‐associated differences in incretin biology and metabolic regulation may influence therapeutic responses to GLP‐1RA. For example, prior studies in adults with obesity have demonstrated higher fasting and stimulated endogenous GLP‐1 concentrations among Black individuals compared with White individuals [28], indicating baseline differences in incretin dynamics that could modify pharmacologic responsiveness to GLP‐1RA therapy. Additionally, while prescription rates of semaglutide were similar between UMMC White and Black populations, tirzepatide, which is associated with the greatest weight loss among GLP‐1RA, was prescribed more often to White patients. This may partially explain the greater weight loss reported in the current study, although the GAMMs controlled for GLP‐1RA type. Differences in insurance coverage of GLP‐1RA may contribute to differences in GLP‐1RA prescription rates [29]. One study revealed that individuals with Medicare or Medicaid were less likely to be on GLP‐1RA as compared with those on private insurance [30]. However, a Veterans Health Administration study revealed that Black individuals in the Veterans Health Administration were more likely to be pushed toward lifestyle interventions and less likely to be prescribed semaglutide as compared to White individuals despite similar insurance coverage [31]. These data highlight disparities in treatment access regardless of insurance coverage. Mississippi is one of only a few states that allows Medicaid coverage of semaglutide and liraglutide (but not tirzepatide) for weight management [32]. Indeed, the lower tirzepatide prescription rates in the Black group were associated with a significantly higher prevalence of Medicaid. Other factors that may also contribute to differences in GLP‐1RA prescription rates include patient preferences, differences in obesity management standards, safety concerns and side effects, and societal and provider bias [29].

While many clinical trials track lifestyle and diet modifications, this information was not captured in the EHR data presented. Thus, a limitation to our findings was the unknown of whether individuals made lifestyle modifications in addition to GLP‐1RA treatment. In addition, only injectable GLP‐1RA were included, while oral formulations were excluded. While dulaglutide is not approved as a primary therapy for weight loss, it has been shown to induce clinically meaningful weight reduction in addition to its glucose lowering effects in individuals with T2D [33]. Given the high prevalence of coexisting obesity and T2D, dulaglutide was included in the analysis to provide a comprehensive evaluation of GLP‐1RA agents associated with weight loss effects. Due to the inconsistency of prescribed dose across each patient, the current study was not able to examine differences between dosages, but recently published data indicate higher doses are associated with greater weight loss [17, 25]. Follow‐up duration varied across patients, reflecting real‐world clinical practice. However, GAMMs handle repeated measures collected at irregular intervals and allow estimation of treatment effects over time without requiring uniform follow‐up windows. Because weight measurements were obtained during routine clinical encounters, Cox regression models do not estimate the time to biologic response, but they estimate the time to first observed documentation of response. Patients with more frequent clinical encounters have more opportunities to meet the event definition earlier, which could bias hazard ratios. Most GLP‐1RA trials report significant decreases in SBP (−5 to −10 mmHg) [17, 21, 23, 34]. However, the current observed early post‐initiation changes may have reflected artifacts from regression to the mean and/or data density. GLP‐1RA discontinuation has been shown to lead to significant weight regain [35, 36]. While we found no differences in discontinuation, one study reported that Black individuals have 8% higher odds of discontinuing GLP‐1RA after 12 months as compared with White individuals [37]. Discontinuation or noncompliance of GLP‐1RA due to adverse effects or insurance status should be considered in future studies. Finally, the present study used EHR data from adult patients in Mississippi and thus may limit the generalizability of our findings to the US population.

## Author Contributions

J.S.C. and J.S.S. contributed to the conception and design of the study. J.H.M., J.S.S., D.M., and J.S.C. drafted the manuscript. J.S.C. and S.T.L. performed statistical analyses. J.H.M., J.S.C., and S.T.L. created all tables and figures. J.H.M., J.S.S., S.T.L., D.M., and J.S.C. revised the manuscript and approved the final manuscript.

## Funding

This work was supported by grants from the National Institute on Minority Health and Health Disparities (R00 MD014738), National Institute of General Medical Sciences (U54 GM115428) to J.S.C., National Heart, Lung, and Blood Institute (U54 HL169191) to J.S.C., National Institute of General Medical Sciences (U54 GM115428 and P30 GM149404) to S.T.L., National Institute of General Medical Sciences (P30 GM149404) to the UMMC Department of Physiology, National Heart, Lung, and Blood Institute (T32 HL105324) to J.H.M. and J.S.S., and National Institute on Diabetes and Digestive and Kidney Diseases (R01 DK124327) to J.S.S.

## Conflicts of Interest

The authors declare no conflicts of interest.

## Supporting information


**Data S1:** oby70180‐sup‐0001‐supinfo.pdf.

## Data Availability

The data that support the findings of this study are available from University of Mississippi Medical Center. Restrictions apply to the availability of these data, which were used under license for this study. Data are available from the author(s) with the permission of University of Mississippi Medical Center.
