# Supplementary material for: Responses to GLP‐1RA in White and Black Adults With Obesity: Insights From Generalized Additive Mixed Models of EHR Data
Source: Obesity (Silver Spring). 2026 Apr 1;34(Suppl 2):109–18. doi: 10.1002/oby.70180 (PMC13191755; doi:10.1002/oby.70180)
Supplement: Supplementary file 1 [file OBY-34-109-s001.pdf]

Online Supplement

Responses to GLP-1RA in White and Black adults with obesity: insights from generalized additive mixed models of EHR data

Jordan H. Mallette[1], Joshua S. Speed[1], Seth T. Lirette[2], Daniel Moore[1], John S. Clemmer[1]

[1]Department of Physiology and Biophysics

University of Mississippi Medical Center

Jackson, MS 39216

[2]Department of Data Science

University of Mississippi Medical Center

Jackson, MS 39216

Address correspondence to:
John S. Clemmer, PhD
Department of Physiology and Biophysics
University of Mississippi Medical Center
2500 North State Street
Jackson, Mississippi 39216-4505
Phone: (601) 984-1802
Email: jclemmer@umc.edu

Supplementary Figure S1. Adjusted smooth effects of continuous covariates in GAMM model of body weight change

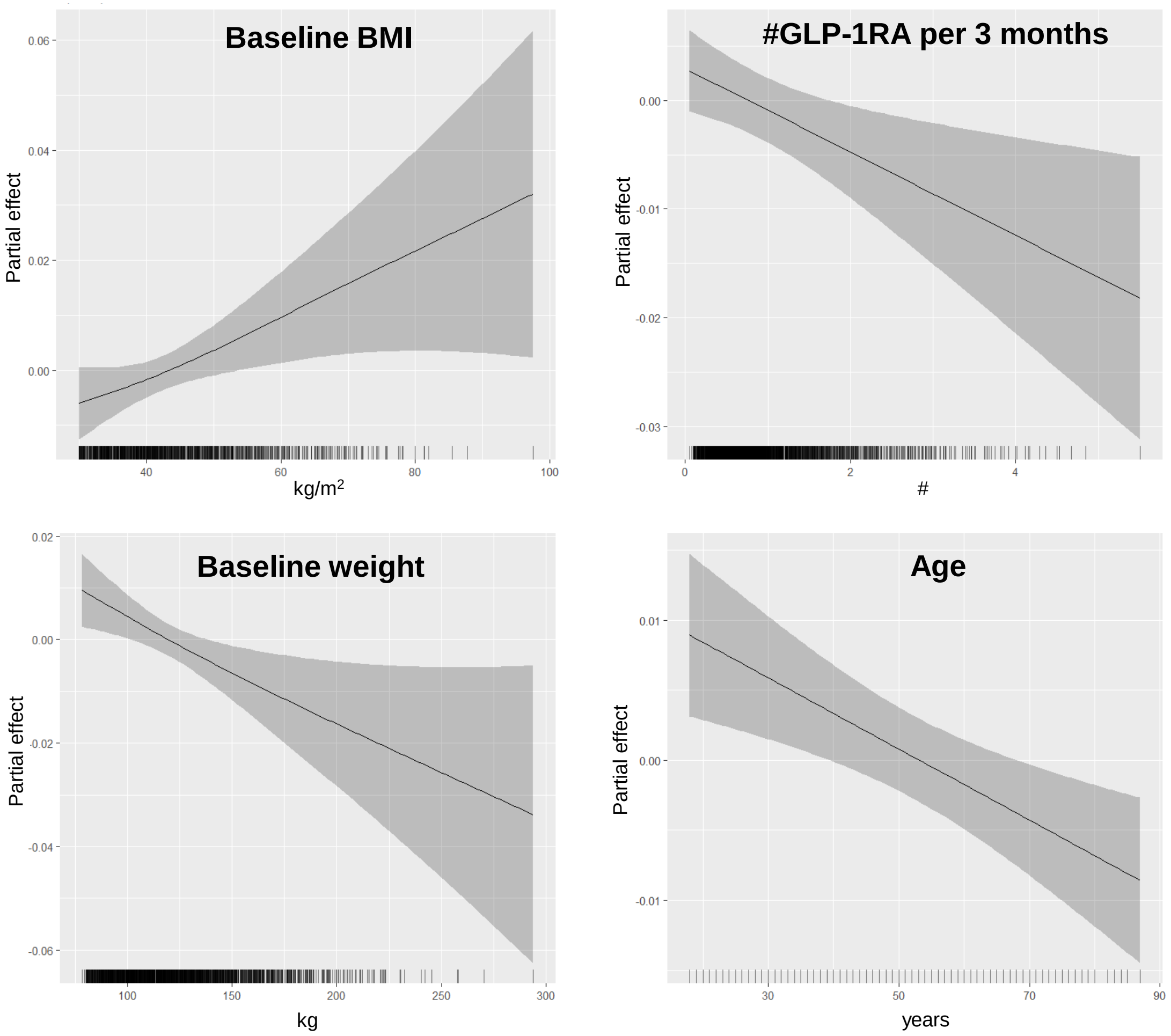

Partial effects of continuous covariates on percent body weight change from generalized additive mixed models (GAMMs) fit to post-treatment data. Each panel displays a smooth function (thin plate spline) for a covariate, adjusted for all other variables in the model. Shaded bands represent 95% confidence intervals. Vertical hash marks (rug plots) along the x-axis indicate the distribution of observed data values used to estimate each smooth term.

Supplementary Figure S2. Adjusted smooth effects of continuous covariates in GAMM model of HbA1C change

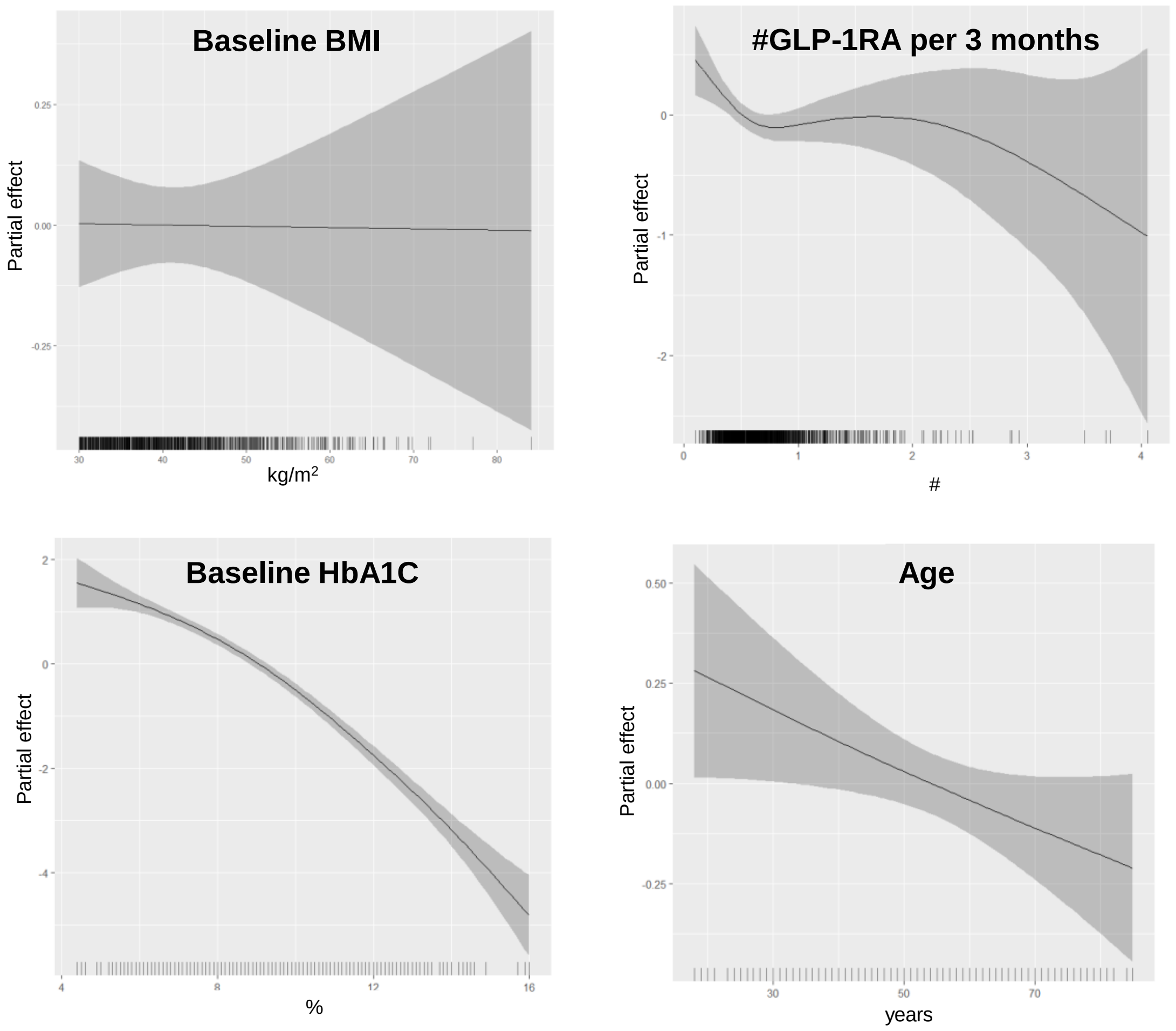

Partial effects of continuous covariates on percent change in HbA1c from generalized additive mixed models (GAMMs) fit to post-treatment data. Each panel shows the estimated smooth function for a covariate, adjusted for other model terms. Shaded regions indicate 95% confidence intervals. Vertical hash marks along the x-axis indicate the distribution of observed data values used to estimate each smooth term.

Supplementary Figure S3. Adjusted smooth effects of continuous covariates in GAMM model of change in systolic blood pressure

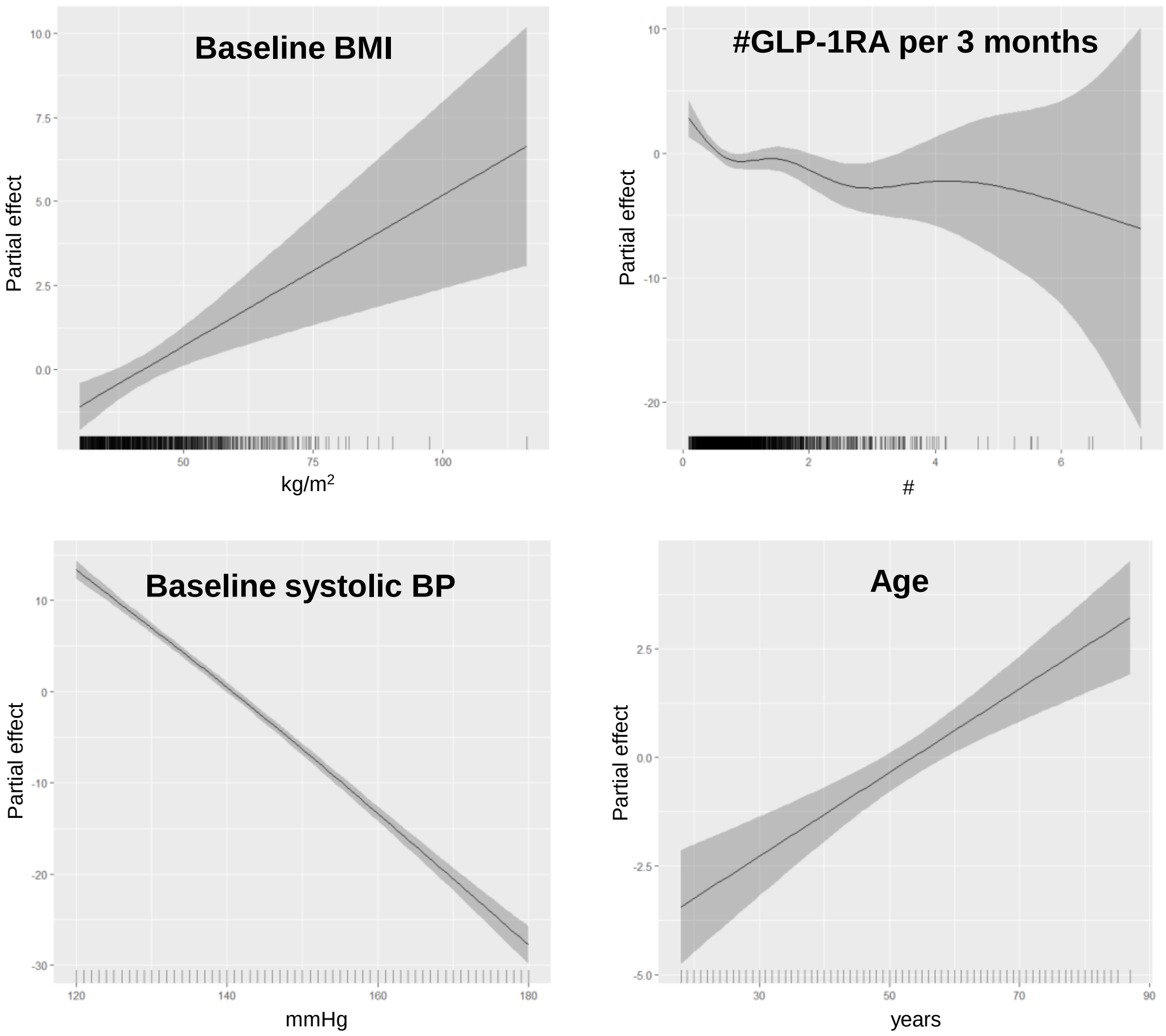

Smooth terms estimated from a GAMM predicting change in systolic blood pressure after GLP-1RA initiation. Partial effects reflect the adjusted association between each continuous covariate and systolic blood pressure change, accounting for other covariates in the model. Thin plate regression splines were used with smoothing estimated via REML; shaded bands represent 95% confidence intervals. Vertical hash marks along the x-axis indicate the distribution of observed data values used to estimate each smooth term.

Supplementary Figure S4. Adjusted smooth effects of continuous covariates in GAMM model of change in eGFR

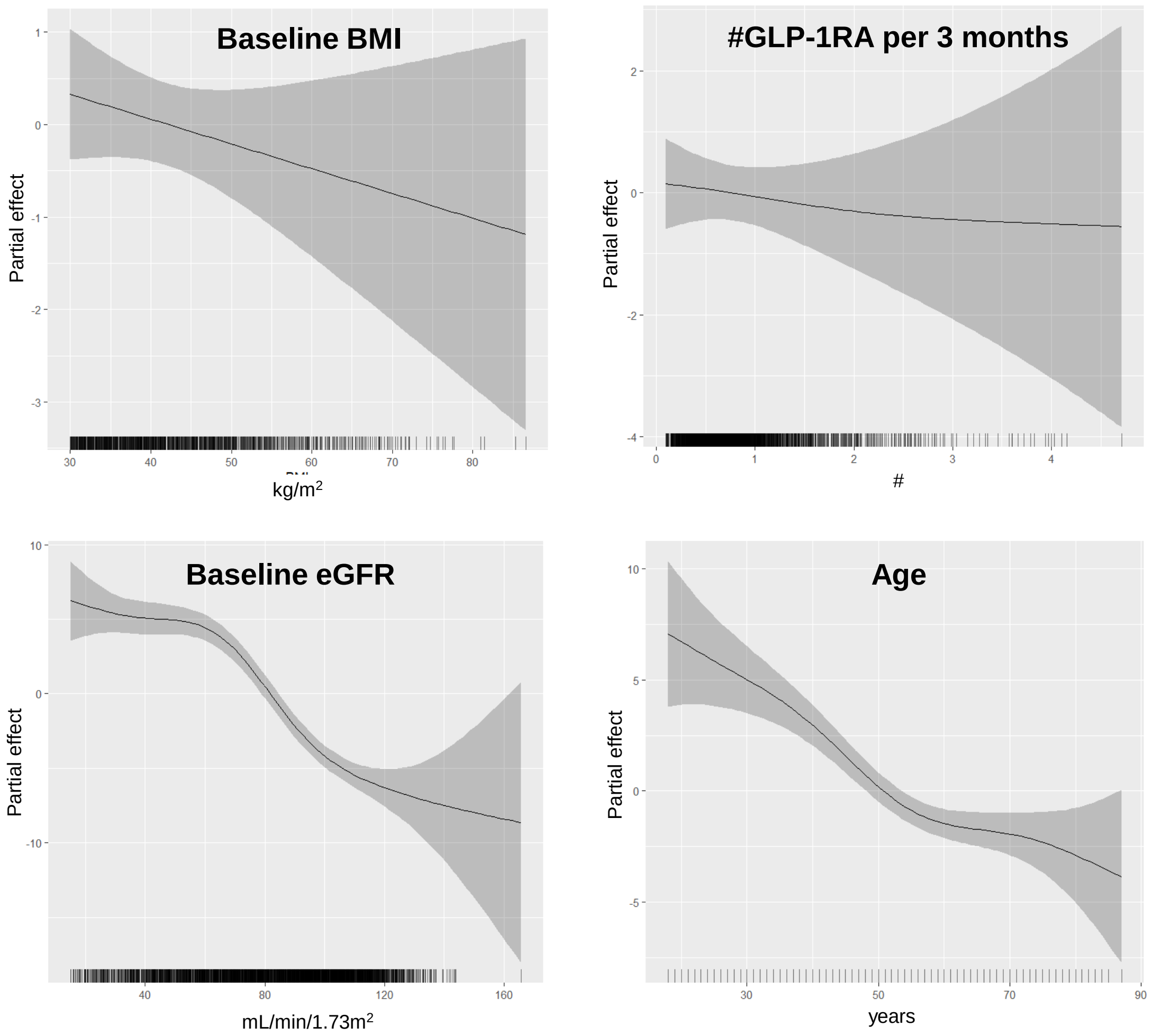

Smooth terms estimated from a GAMM predicting change in estimated glomerular filtration rate (eGFR) after GLP-1RA. Partial effects reflect the adjusted association between each continuous covariate and eGFR change. Thin plate regression splines were used with smoothing estimated via REML; shaded bands represent 95% confidence intervals. Vertical hash marks along the x-axis indicate the distribution of observed data values used to estimate each smooth term.

Supplementary Table S1. SNOMED-CT codes used to define baseline comorbidities

Condition	SNOMED-CT Concept	SNOMED-CT Concept ID
Type 2 diabetes mellitus	Type 2 diabetes mellitus	44054006
Hypertension	Hypertensive disorder, systemic arterial	38341003
Dyslipidemia	Disorder of lipid metabolism	55822004
Coronary artery disease	Coronary arteriosclerosis	53741008
Acute kidney injury	Acute kidney injury	14669001
Chronic kidney disease	Chronic kidney disease	709044004
End-stage renal disease	End-stage renal disease	90688005
Heart failure	Heart failure	84114007
Myocardial infarction	Myocardial infarction	22298006

Supplemental Table S2. Parametric Coefficients from Generalized Additive Mixed Models of Longitudinal Changes after GLP-1RA Initiation

Change in Body Weight (%)

Parametric coefficient (β)	Estimate	SE	p-value
(Intercept)	-0.0595	0.0044	<0.0001
Male sex	0.0132	0.0026	<0.0001
Black race	0.0174	0.0036	<0.0001
Diabetes	0.0057	0.0027	0.033
Medicaid	0.0032	0.002	0.114
Liraglutide	-0.0065	0.0027	0.015
Semaglutide	-0.0106	0.0023	<0.0001
Tirzepatide	-0.0124	0.0038	0.0009

Change in HbA1c (%)

Parametric coefficient	Estimate	SE	P value
(Intercept)	-1.026	0.115	<0.0001
Male sex	-0.037	0.091	0.683
Black race	0.211	0.098	0.031
Liraglutide	0.014	0.104	0.891
Semaglutide	-0.271	0.098	0.0056
Tirzepatide	-0.351	0.175	0.044
Medicaid	0.282	0.113	0.012

Change in Systolic Blood Pressure (mmHg)

Parametric coefficient	Estimate	SE	P value
(Intercept)	-8.43	0.88	<0.0001
Male sex	1.8	0.48	0.0002
Black race	3.24	0.52	<0.0001
Diabetes	1.29	0.63	0.040
Liraglutide	-0.05	0.64	0.94
Semaglutide	-2.03	0.57	0.00036
Tirzepatide	-2.06	0.84	0.014
Medicaid	-0.42	0.49	0.393

Change in eGFR (mL/min/1.73 m²)

Parametric coefficient	Estimate	SE	P value
(Intercept)	-1.93	0.91	0.035
Male sex	-0.27	0.47	0.567
Black race	-1.72	0.53	0.0012
Diabetes	-0.56	0.71	0.428
Liraglutide	-0.43	0.61	0.478
Semaglutide	0.59	0.54	0.273
Tirzepatide	0.21	0.86	0.809
Medicaid	-1.27	0.45	0.0049

Models were estimated using generalized additive mixed models with a smooth term for time after GLP-1RA initiation and random intercepts for individuals. All models were adjusted for age, sex, race, diabetes status, Medicaid enrollment, BMI, baseline values, and GLP-1RA frequency and formulation. Dulaglutide served as the reference category for GLP-1RA type. Estimates represent average population-level effects across the follow-up period. P values are two-sided.

Supplemental Table S3. Predicted mean percent change in body weight by race and GLP-1RA formulation over follow-up

	%Change in body weight		
**White**	1 year	2.5 years	5 years
Dulaglutide	-0.050 (-0.056, -0.043)	-0.069 (-0.079, -0.059)	-0.115 (-0.136, -0.095)
Liraglutide	-0.055 (-0.061, -0.049)	-0.074 (-0.084, -0.064)	-0.121 (-0.141, -0.100)
Semaglutide	-0.060 (-0.065, -0.055)	-0.079 (-0.089, -0.070)	-0.126 (-0.146, -0.106)
Tirzepatide	-0.063 (-0.071, -0.055)	-0.082 (-0.093, -0.071)	-0.129 (-0.149, -0.108)
**Black**	1 year	2.5 years	5 years
Dulaglutide	-0.035 (-0.039, -0.030)	-0.047 (-0.053, -0.040)	-0.095 (-0.107, -0.083)
Liraglutide	-0.040 (-0.045, -0.035)	-0.052 (-0.058, -0.045)	-0.100 (-0.112, -0.088)
Semaglutide	-0.045 (-0.049, -0.041)	-0.057 (-0.063, -0.051)	-0.105 (-0.117, -0.093)
Tirzepatide	-0.048 (-0.055, -0.042)	-0.060 (-0.068, -0.052)	-0.108 (-0.121, -0.095)

Values are adjusted marginal means with 95% confidence intervals estimated from generalized additive mixed models (GAMMs). Estimates were evaluated fixing race or GLP1-RA formulation and other covariates at reference values. The model was adjusted for age, sex, race, diabetes, Medicaid enrollment, BMI, baseline body weight, and GLP-1RA frequency and formulation.

Supplemental Table S4. Predicted mean change in eGFR (mL/min/m^2) by race and GLP-1RA formulation over follow-up

	eGFR (mL/min/m^2)		
**White**	1 year	2.5 years	5 years
Dulaglutide	-1.326 (-2.787, 0.134)	-3.275 (-4.921, -1.629)	-6.523 (-8.961, -4.084)
Liraglutide	-1.745 (-3.170, -0.320)	-3.693 (-5.308, -2.078)	-6.941 (-9.359, -4.523)
Semaglutide	-0.727 (-2.002, 0.548)	-2.676 (-4.157, -1.194)	-5.923 (-8.252, -3.595)
Tirzepatide	-1.104 (-2.918, 0.710)	-3.053 (-5.021, -1.085)	-6.301 (-8.969, -3.632)
**Black**	1 year	2.5 years	5 years
Dulaglutide	-2.833 (-4.090, -1.576)	-5.238 (-6.561, -3.915)	-9.247 (-10.923, -7.570)
Liraglutide	-3.251 (-4.507, -1.996)	-5.657 (-6.980, -4.333)	-9.665 (-11.344, -7.987)
Semaglutide	-2.234 (-3.291, -1.176)	-4.639 (-5.776, -3.502)	-8.648 (-10.184, -7.112)
Tirzepatide	-2.611 (-4.275, -0.947)	-5.016 (-6.729, -3.304)	-9.025 (-11.020, -7.029)

Values are adjusted marginal means with 95% confidence intervals estimated from generalized additive mixed models (GAMMs). Estimates were evaluated fixing race or GLP1-RA formulation and other covariates at reference values. The model was adjusted for age, sex, race, diabetes, Medicaid enrollment, BMI, baseline eGFR, and GLP-1RA frequency and formulation.

Supplemental Table S5. Predicted mean change in blood pressure by race and GLP-1RA formulation over follow-up

	Change in systolic blood pressure (mmHg)		
**White**	1 year	2.5 years	5 years
Dulaglutide	-7.010 (-8.383, -5.638)	-7.987 (-9.535, -6.440)	-9.615 (-12.005, -7.225)
Liraglutide	-7.062 (-8.367, -5.758)	-8.039 (-9.527, -6.551)	-9.667 (-12.020, -7.315)
Semaglutide	-9.038 (-10.177, -7.898)	-10.015 (-11.358, -8.672)	-11.643 (-13.904, -9.381)
Tirzepatide	-9.073 (-10.720, -7.426)	-10.050 (-11.843, -8.257)	-11.678 (-14.231, -9.124)
**Black**	1 year	2.5 years	5 years
Dulaglutide	-4.158 (-5.316, -3.000)	-4.270 (-5.511, -3.028)	-7.345 (-9.467, -5.223)
Liraglutide	-4.210 (-5.348, -3.072)	-4.322 (-5.548, -3.096)	-7.397 (-9.511, -5.284)
Semaglutide	-6.186 (-7.102, -5.269)	-6.297 (-7.326, -5.269)	-9.373 (-11.380, -7.365)
Tirzepatide	-6.221 (-7.737, -4.704)	-6.332 (-7.914, -4.751)	-9.408 (-11.747, -7.069)

Values are adjusted marginal means with 95% confidence intervals estimated from generalized additive mixed models (GAMMs). Estimates were evaluated fixing race or GLP1-RA formulation and other covariates at reference values. The model was adjusted for age, sex, race, diabetes, Medicaid enrollment, BMI, baseline body weight, and GLP-1RA frequency and formulation.

Supplemental Table S6. Predicted mean change in HbA1c by race and GLP-1RA formulation over follow-up

	Change in HbA1c		
**White**	1 year	2.5 years	5 years
Dulaglutide	-0.964 (-1.220, -0.708)	-1.045 (-1.344, -0.746)	-1.599 (-2.284, -0.913)
Liraglutide	-0.950 (-1.201, -0.698)	-1.031 (-1.326, -0.736)	-1.584 (-2.268, -0.901)
Semaglutide	-1.235 (-1.472, -0.998)	-1.316 (-1.601, -1.032)	-1.870 (-2.549, -1.190)
Tirzepatide	-1.315 (-1.685, -0.944)	-1.396 (-1.797, -0.995)	-1.949 (-2.683, -1.215)
**Black**	1 year	2.5 years	5 years
Dulaglutide	-0.850 (-1.056, -0.643)	-0.732 (-0.958, -0.505)	-0.935 (-1.518, -0.353)
Liraglutide	-0.836 (-1.049, -0.622)	-0.717 (-0.950, -0.485)	-0.921 (-1.506, -0.336)
Semaglutide	-1.121 (-1.311, -0.931)	-1.003 (-1.216, -0.790)	-1.206 (-1.783, -0.630)
Tirzepatide	-1.201 (-1.544, -0.857)	-1.083 (-1.437, -0.728)	-1.286 (-1.929, -0.644)

Values are adjusted marginal means with 95% confidence intervals estimated from generalized additive mixed models (GAMMs). Estimates were evaluated fixing race or GLP1-RA formulation and other covariates at reference values. The model was adjusted for age, sex, race, diabetes, Medicaid enrollment, BMI, baseline HbA1c, and GLP-1RA frequency and formulation.
